# Transgene and Chemical Transdifferentiation of Somatic Cells for Rapid and Efficient Neurological Disease Cell Models

**DOI:** 10.3389/fncel.2022.858432

**Published:** 2022-05-11

**Authors:** Neville Ng, Michelle Newbery, Simon Maksour, Mirella Dottori, Ronald Sluyter, Lezanne Ooi

**Affiliations:** ^1^Illawarra Health and Medical Research Institute, Wollongong, NSW, Australia; ^2^School of Chemistry and Molecular Bioscience and Molecular Horizons, University of Wollongong, Wollongong, NSW, Australia; ^3^School of Medical, Indigenous and Health Sciences, University of Wollongong, Wollongong, NSW, Australia

**Keywords:** transdifferentiation, reprogramming, differentiation, neurodegeneration, transcription factors, aging

## Abstract

For neurological diseases, molecular and cellular research relies on the use of model systems to investigate disease processes and test potential therapeutics. The last decade has witnessed an increase in the number of studies using induced pluripotent stem cells to generate disease relevant cell types from patients. The reprogramming process permits the generation of a large number of cells but is potentially disadvantaged by introducing variability in clonal lines and the removal of phenotypes of aging, which are critical to understand neurodegenerative diseases. An under-utilized approach to disease modeling involves the transdifferentiation of aged cells from patients, such as fibroblasts or blood cells, into various neural cell types. In this review we discuss techniques used for rapid and efficient direct conversion to neural cell types. We examine the limitations and future perspectives of this rapidly advancing field that could improve neurological disease modeling and drug discovery.

## Introduction

Transdifferentiation involves the conversion of one mature somatic cell type into another mature somatic cell type, without transition through a pluripotent stage. As such, transdifferentiation may involve driving gene expression programs by ectopic transcription factor expression and/or the use of growth factors and chemicals to promote differentiation into a specific cell type. Understanding the control of cell fate switches is becoming increasingly important in the field of disease modeling, particularly for diseases of aging and for cell types that are difficult to obtain *via* biopsy. Consequently, neurological research will benefit from developments in transdifferentiation approaches. However, caution should be applied in the interpretation of data using these cells for a variety of reasons. The following mini-review outlines transdifferentiation methods, as well as strengths and limitations pertinent to this rapidly advancing field.

Patient live cell-based assay platforms, combined with exogenous stressors/stimuli to emulate disease factors, offer an effective avenue to investigate disease or drug mechanisms, with human relevance and without animal experimentation. Understanding neurological disease factors across a myriad of genetic or environmental factors with larger scale human population studies not only assists distinguishing causative factors from untreatable hallmarks, but also accounts for inter-individual variability and pharmacogenetic factors. The advent of routine reprogramming of somatic cells into induced pluripotent stem cells (iPSCs), using lentiviral, transgene-free and cDNA-based protocols, has enabled the routine conversion of non-invasively acquired cells *via* pluripotent stem cells to cell types that are not otherwise readily accessible. Advances in efficient microplate, microfluidic (Beers et al., 2015; Gagliano et al., 2019) reprogramming and cost-effective maintenance methods (Kuo et al., 2020) can reduce the significant cost and time demand of iPSC generation and handling. However, variability can arise from iPSC lines as a result of selection and handling (Volpato and Webber, 2020), while growth factor differentiation methods can vary in outcomes with cell culture density (Tonge and Andrews, 2010; Julia et al., 2017). Experiments using panels of cell lines can thus cause variation in differentiation outcomes, which can lead to inconsistencies in downstream assays (Volpato and Webber, 2020).

## Recent Advances in Transdifferentiation of Fibroblasts or Blood Cells to Neurological Disease Relevant Cell Types

**FIGURE 1:**
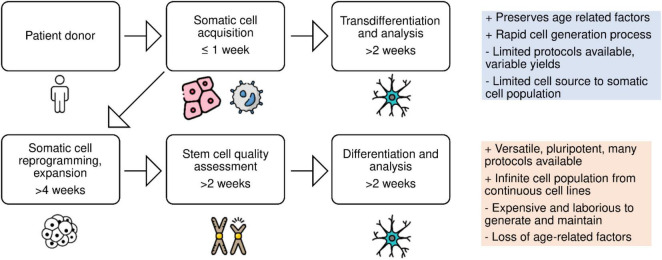
Schematic of patient-derived cell reprogramming. Neural cell types can be generated *via* reprogramming somatic cells into induced pluripotent stem cells, followed by differentiation, or *via* direct conversion of somatic cells into neural cell types, bypassing the stem cell stage.

Complementary to reprogramming of somatic cells, the transdifferentiation of human patient skin or blood biopsy samples offers a simple means to generate neurologically relevant cell types (Figure 1).

Transdifferentiation has previously been carried out using fibroblasts or peripheral blood mononuclear cells (PBMCs) to neural stem cells, neurons, astrocytes, oligodendrocytes, microglia, and skeletal muscle cells (Table 1). Direct differentiation is not limited to a terminally differentiated endpoint; for neurogenesis, reprogramming of fibroblasts to multipotent neural precursor cells can be performed with the overexpression of transcription factors SOX2, OCT3, KLF4, and MYC (Meyer et al., 2014), SOX2 and PAX6 (Maucksch et al., 2012), or even a single transcription factor, such as PTF1A (Xiao et al., 2018).

**TABLE 1:** Proof of concept advances in human or animal-derived direct conversion to neurological disease relevant cell type.

Differentiation cell type	References	Source cell	Transdifferentiation factors		Differentiation time (yield)	Functional characterization
			Transgenes	Chemical compounds		
Neural stem cell	Han et al., 2012	MEF	BRN4/POU3F4, SOX2, KLF4, and MYC	N/A	4 weeks	Multipotency
	Meyer et al., 2014	HDF	SOX2, KLF4, OCT3, c-Myc	N/A	6–10 days (60–95%)	Multipotency
	Cheng et al., 2014	MEF, MTTF	N/A	Valproic acid, CHIR99021 and RepSox	12 days (∼22%)	Multipotency
	Lee et al., 2015	Human neonatal cord blood and adult peripheral blood	OCT4	SB431542, LDN-193189, Noggin and CHIR99021	10–14 days	Multipotency and self-renewal capacity
	Tang et al., 2016	Human PBMC	OCT4, SOX2, NANOG, LIN28, c-Myc and KLF4	LIF, CHIR99021 and SB431542	30 days	Multipotency and self-renewal capacity
	Sheng et al., 2018	Human PBMC	SOX2 and c-MYC,	CHIR99021, purmorphamine, A83-01, LIF and tranylcypromine	10–21 days	Multipotency and self-renewal capacity
	Thier et al., 2019	Human PBMC, HFPF and HDF	BRN2, SOX2, KLF4 and ZIC3	CHIR99021, purmorphamine and tranylcypromine	19–24 days	Multipotency and self-renewal capacity
	Kim et al., 2018	UCB-MSC	SOX2	N/A	14 days	Multipotency and self-renewal capacity
	Xiao et al., 2018	HFF	PTF1A	N/A	9–14 days	Multipotency
Neuron	Liu et al., 2013	HLF, HDF	NGN2	Dorsomorphin and forskolin	1–2 weeks (∼90%)	Voltage and current clamp
	Hu et al., 2015	HFF	N/A	Valproic acid, CHIR99021, RepSox, forskolin, SP600625, GO6983, Y-27632 and dorsomorphin	3–4 weeks, (>80%)	Whole cell patch clamp
	Victor et al., 2018	HDF	miR-9/9* and miR-124 fused to Bcl-xL, CTIP2, MYT1L, DLX1 and DLX2	dbCAMP, valproic acid and retinoic acid	4–5 weeks (90%)	Whole cell patch clamp
	Tanabe et al., 2018	Human PBMC or T-lymphocytes	BRN2, ASCL1, MYT1 and NGN2	Forskolin, dorsomorphin, SB431542 and Y-27632	3–7 weeks	Whole cell patch clamp
	Yang et al., 2019	HFF, HDF	P7C3 and ISX9	Purmorphamine, dorsomorphin, CHIR99021, forskolin, LDN193189, RG108, PD0325901, A83-01, DAPT and Y-27632	10 days (∼85%)	Whole cell patch clamp
Motor neuron	Son et al., 2011	HEF	HB9, ISL1, LHX3, ASCL1, BRN2, MYT1L, NGN2	N/A	10 days	Whole cell patch clamp
	Lee et al., 2020	HDF	POU5F1, LHX3	N/A	24 days, (> 96%)	Whole cell patch clamp, *in vivo* mouse transplant
Astrocyte	Caiazzo et al., 2015	MEF, MDF	NFIA, NFIB, SOX9	N/A	2 weeks, (∼15%)	Voltage gated potassium current, inflammatory cytokine secretion, glutamate uptake
Oligodendrocyte	Yang et al., 2013	MEF, REF	SOX10, OLIG2, Zfp536	Progesterone, D-biotin, forskolin, PDGF-AA and NT-3	3 weeks, (∼16%)	Myelination in coculture and mouse transplant
	Najm et al., 2013	MEF, MLF	SOX10, OLIG2, NKX6.2	SHH and PDGF-AA	3 weeks, (∼21%)	Myelination in mouse transplant
	Kim et al., 2015	MDF	OCT4	PDGF-AA and FGF2	4 weeks	Myelination in mouse transplant
	Chanoumidou et al., 2021	HDF, human fetal lung fibroblasts	SOX10, OLIG2, NKX6.2	SAG, PDGF, Ascorbic acid T3, NT-3, IGF-1 and dbcAMP	2–3 weeks (39.1%)	Myelination *in vitro*, oligodendrocytes in Pelizaeus-Merzbacher disease model
Microglia	Ohgidani et al., 2014	PBMC	N/A	GM-CSF, IL-34 and M-CSF	2 weeks	Phagocytosis, cytokine secretion
	Banerjee et al., 2021	PBMC	N/A	GM-CSF and IL-34	2 weeks	Phagocytosis, microglia marker expression, RNA sequencing
Skeletal muscle	Kim et al., 2016	Human urine-derived cells	MYOD	Horse serum, hydrocortisone and dexamethosone	1–3 weeks (80%)	Skeletal muscle in Duchenne muscular dystrophy model
	Ito et al., 2017	MEF/MTTF	PAX7, MYOD, MEF2B	Chick embryo extract, FGF2 and LIF	2 weeks	Skeletal muscle regeneration in Duchenne muscular dystrophy model
	Wakao et al., 2017	HDF	MYOD, MYCL	Horse serum and IGF-1	2 weeks (∼ 90%)	Muscle fiber formation in mouse model
	Bar-Nur et al., 2018	MEF	MYOD	Horse serum, forskolin, RepSox and CHIR99021	2–3 weeks	Skeletal muscle regeneration in mouse injury model

*MEF, murine embryonic fibroblast. MDF, murine dermal fibroblast. MLF, murine lung fibroblast. MTTF, mouse tail tip fibroblast. HEF, human embryonic fibroblast. HDF, human dermal fibroblast. HFF, human foreskin fibroblast. HFPF, human fetal pancreas fibroblasts. REF, rat embryonic fibroblast. PBMC, peripheral blood mononuclear cell. UCB-MSC, umbilical cord blood derived-mesenchymal stem cell.*

Along with many studies utilizing iPSCs in recent years as a patient derived disease modeling source, are studies indicating that the reprogramming process and subsequent clonal selection erases age-accumulated disease phenotypes, thus complicating their comparison with patient tissue studies (Mertens et al., 2018). An advantage of direct conversion methods is the retainment of age-accumulated factors, such as epigenetic DNA methylation, DNA damage lesions, heterochromatin organization, senescence associated β-galactosidase activity, and proteostasis, which are re-set when reprogramming cells to a pluripotent state (Mertens et al., 2015, 2018; Tang et al., 2017). Additionally, the reprogramming process results in heterogenous telomerase activation (Huang et al., 2011; Allsopp, 2012).

Tang et al. (2017) assessed age-related cellular hallmarks in motor neurons transdifferentiated from old and young human dermal fibroblasts, in comparison to motor neurons derived from iPSCs. The method of motor neuron differentiation from iPSCs was the same as used for transdifferentiating human fibroblasts, i.e., exogenous expression of the motor neuron-specifying factors, neurogenin-2 (NGN2), SOX11, islet-1 (ISL1), and LIM homeobox 3 (LHX3). Markers of heterochromatin and nuclear organization (LAP2α, H3K9m3 and HP1γ) were found to be reduced in induced motor neurons derived from the older fibroblasts, relative to motor neurons derived from younger fibroblasts or iPSCs. Another study using donors ranging from 3 days old to 96 years old, demonstrated that conversion of fibroblast samples to neurons using neuronal microRNAs (miRNAs) retained numerous age-associated features, including epigenetic CpG loci methylation signatures, elevated oxidative stress, DNA damage and telomere shortening (Huh et al., 2016). Similarly, age-associated factors observed in induced neurons from fibroblasts from aged donors but not iPSCs include increased DSB repair lesions (elevated γH2AX foci), and dysfunctional nuclear pore complex transport with poor nuclear-cytoplasmic localization and export correlated with loss of RanBP17 expression (Mertens et al., 2018). Striatal neurons generated by miRNA-based direct neuronal conversion of Huntington’s disease patient dermal fibroblasts retained age related phenotypes and spontaneously formed nuclear huntingtin protein aggregates, whereas neurons from iPSCs were aggregate-free (Victor et al., 2018). Given that many neurodegenerative disease phenotypes emerge with aging, the approach of transdifferentiation is complementary with reprogramming for the purposes of neurological disease modeling.

The transdifferentiation of neural cells from patient blood cells affords similar investigative opportunities as iPSC-derived neural cells, including the study of neurological mechanisms in health and disease, drug screening and neurodevelopment (Li L. et al., 2018). Multiple laboratories have demonstrated the potential to transdifferentiate human blood cells into various neural cell types, including neurons, microglia, astrocytes and oligodendrocytes, which may also facilitate the development and study of more complex 3D culture models, in addition to the study of individual neural cell types or co-cultures of different cell types. One seminal study reported the generation of CD34^+^ neural progenitor cells from adult peripheral blood and cord blood, which could be subsequently differentiated into dopaminergic or nociceptive neurons, astrocytes or, in very limited cases, oligodendrocytes (Lee et al., 2015). The functional characterization of either glial subtype was not explored in this study. However, the nociceptive neurons were used to develop a model of chemotherapy-induced neuropathy suitable for drug screening. Moreover, human adult peripheral blood T cells, following IL-2 and CD3/CD28 receptor stimulation, can be transdifferentiated directly into neurons, which bypassed the need to generate neural progenitor cells and which were capable of forming action potentials and immature but functional synapses (Tanabe et al., 2018). IL-2 and CD3 receptor stimulation has also been used to expand human adult PBMCs that can be transdifferentiated into photoreceptor-like cells (Komuta et al., 2016), allowing for future study of the retina and visual neurosensory system. In relation to glia, human adult PBMCs can be transdifferentiated into microglia with an immune phenotype characteristic of resident microglia, including phagocytic activity and tumor necrosis factor-α release (Ohgidani et al., 2014). Notably, this study revealed that microglia transdifferentiated from monocytes from a patient with Nasu-Hakola disease had an altered inflammatory cytokine profile, compared to microglia from a healthy subject, highlighting the potential of this approach to study microglial phenotypes in neurological disorders. The possibility remains that astrocytes can also be transdifferentiated from human blood cells. A novel population of CD45^+^ peripheral blood cells, termed insulin-producing cells, can give rise to astrocyte-like cells (Li et al., 2015) but the functional activity of these cells is yet to be reported. Nevertheless, this study is reminiscent of early studies with human blood monocyte-derived microglia (Etemad et al., 2012; Noto et al., 2014) that contributed to the aforementioned transdifferentiation of microglia from monocytes (Ohgidani et al., 2014). Finally, human CD34^+^ cells, obtained from granulocyte colony stimulating factor (GM-CSF)-mobilized peripheral blood, can be transdifferentiated to oligodendrocytes (Venkatesh et al., 2015). Although functional characterization of these cells was lacking, the future development of such cells could be considered as a transplant option to remyelinate neurons in neurological disorders, such as multiple sclerosis, as well as in spinal cord injuries. These studies demonstrate the utility of blood cells for neural cell generation for neurological disease modeling.

## Approaches to Transdifferentiation Using Chemicals and Transcription Factors

Chemical transdifferentiation methods offer a simple approach for modulation of relevant pathways for direct conversion. Commonly antagonized pathways for enhancement of neuronal differentiation include the bone morphogenetic protein (BMP) pathway, (e.g., commonly used inhibitors dorsomorphin, LDN193189), pro-proliferative Notch pathway (e.g., DAPT), TGF-β signaling (e.g., RepSox), and inhibition of GSK-3 kinase activity leading to WNT activation (e.g., CHIR99021), often combined with elevation of cAMP levels (e.g., dibutyryl-cAMP, forskolin) (Crawford and Roelink, 2007; Hu et al., 2015). Such cocktails have been demonstrated to convert human fibroblasts to functional forebrain glutamatergic neurons within 2 weeks (Yang et al., 2019). PBMCs have been demonstrated to transdifferentiate to functional microglia within 2 weeks in the presence of the cytokines, GM-CSF and interleukin (IL)-34 (Ohgidani et al., 2014).

Transcription factor over-expression methods typically include transgene delivery by plasmid cDNA, membrane permeable factor conjugated protein (e.g., human immunodeficiency virus-1 *trans*-activator of transcription, TAT) (Pan et al., 2010) or viral transduction. Transgenic over-expression enables directed subtype specification, as opposed to retrospective identification of a differentiated subtype, such as the specification of cholinergic motor neurons with the transcription factor NGN2, in combination with motor neuron transcription factors, ISL1 and LHX3 (Fernandopulle et al., 2018). Over-expression of transcription factors, SRY-box transcription factor 9 (SOX9), nuclear factor IA (NFIA) and NFIB have been investigated for their capacity to differentiate stem cells to astrocytes (Canals et al., 2018; Li X. et al., 2018), as well as direct transdifferentiation from murine fibroblasts (Caiazzo et al., 2015) within 2–4 weeks. To facilitate transgene over-expression differentiation protocols, lentiviral particle delivery systems can be cost effective and simple to set up and perform routinely in basic cell culture laboratories (Newbery et al., 2021). An added advantage of viral transduction-based methods is that poorly transduced high density cell clusters that may provide a source of heterogeneity in a non-selective differentiation protocol are removed following antibiotic selection. However, in application ineffective antibiotic selection can result in proliferative non-transduced cell populations. These issues may be overcome by increasing antibiotic titer or utilizing an alternative antibiotic. In combination with transcription factor over-expression, differentiation methods can also be modulated with small molecules to improve maturity or alter non-homogenous culture outcomes. For example dibutyryl-cAMP (Taylor and Reh, 1990; Vidaltamayo et al., 1996) and DAPT (Crawford and Roelink, 2007) are often included in NGN2-based neuronal differentiation protocols (Zhang et al., 2013; De Santis et al., 2018) given their effect on yield and maturity. Cytotoxic additives, such as cytosine arabinoside or CultureOne can also be included in neuronal differentiations to remove proliferating neural/glial progenitors from a post-mitotic neuronal population (Wallace and Johnson, 1989; Fernandopulle et al., 2018). The addition of BMP pathway inhibition (e.g., dorsomorphin or LDN193189) to neuronal differentiation medium could also potentially influence the fate of “bystander” glial cells from an astrocytic lineage to oligodendrocyte lineage for co-culture studies (Hu et al., 2010).

Although lentiviral vectors can be simple and cost effective to produce, the possibility of an integration-induced phenotype limits their clinical application. Furthermore forced exogenous transcription factor overexpression could also induce artifactual epigenetic signatures (Mollinari and Merlo, 2021). Use of small molecules alone may circumvent these limitations, while the cost of small molecules can become a prohibitive factor to efficient scalability. Furthermore, identification of novel small molecules to replace exogenous factor expression is challenging given their indirect control of transcription factors that control lineage (Mertens et al., 2018). The application of non-viral vectors for direct conversion of fibroblasts has been demonstrated in alternative methods involving polysaccharide cationic polymers, electroporation, and combinations of lipid, polycation and magnetofection, however, these non-viral methods remain challenged with low efficiencies (Gong et al., 2018).

## Discussion: Limitations and Future Perspectives

Neurological disease relevant transdifferentiation protocols over the past decade include conversion of fibroblasts or PBMCs to multipotent neural stem cells or functional neurons, astrocytes, microglia, oligodendrocytes and skeletal muscle. Retaining potential age-associated neurological disease factors, these models can be implemented to complement reprogrammed somatic cell studies that explore disease modeling from nascent pluripotent stages. However, transdifferentiation approaches are not without practical setbacks, largely owing to the limited protocols available for specific differentiation outcomes and practical yield.

Similar to the considerations pertinent to the reprogramming of somatic cells to iPSCs, PBMCs are readily accessible from blood, however, have much lower proliferative potential than the more invasively acquired dermal fibroblasts (Panda and Ravindran, 2013). Access to a proliferative dermal cell population may be feasible *via* hair follicle multipotent neural crest stem cells (Gho et al., 2016; Molina and Finol, 2020). The direct conversion of PBMCs is challenged by yield and low proliferative capacity, for example the transdifferentiation of blood to functional neurons produced a modest yield of 50,000 cells per mL (∼3% conversion rate) (Tanabe et al., 2018). In either case, for studies requiring larger cell populations, it may be more efficient to generate progenitor populations from PBMCs or dermal fibroblasts (Meyer et al., 2014; Lee et al., 2015). The advantage of generating progenitors is that they can be expanded and frozen, which may provide a more efficient and long-term resource for generating differentiated cell types, rather than relying on limited stocks of primary cells.

Challenges in neurological cell-based models (not limited to transdifferentiation protocols), include homogeneity, maturity and subtype specification. Neuronal differentiation by NGN2 over-expression has been observed to generate homogenous MAP2^+^ neuronal populations, but generates neurons with mixed gene expression signatures of central and peripheral nervous system markers that differ depending on duration of over-expression (Lin et al., 2021), while synaptic formation is known to be limited when transdifferentiating from fibroblasts, as opposed to iPSCs or neural precursor cells (Zhang et al., 2013). Microarray analysis of SOX9, NFIA and NFIB astrocytic transdifferentiated fibroblasts show similarities to primary cortical astrocytes, relative to fibroblasts, but with many distinct heterogeneous populations. Additionally, as with many transdifferentiation protocols, further investigation is required to identify the precise anatomical region these cells represent and their maturity, compared to astrocytes in human tissue (Caiazzo et al., 2015).

### Co-culture and 3D Modeling

Direct conversion methods are a logical approach for human drug and disease studies in mono-culture and co-culture formats. Co-culture models may improve our understanding of intercellular interactions, such as neurotoxic inflammation by activated glial cells, demyelination processes or neuromuscular junction formation. Neuronal-glial co-cultures may be utilized to emulate an exogenous source of stress (e.g., viral, inflammatory, protein aggregate, etc.), with subsequent glial cell dysfunction or activation, and the resultant effects on neuronal cell function or survival. A distinct advantage of direct differentiation methods is the rapid implementation of live cell culture models. The transdifferentiation of somatic cells to neural progenitor cells can also be utilized to support organoid generation, on the basis of initiating growth from extrinsic-patterned cultures rather than stem cells (Pacitti et al., 2019). For the purposes of drug testing, assessing inter-individual differences in absorption, distribution, metabolism and excretion (ADME), renal toxicity, hepatotoxicity and CYP450 inhibition assays are currently feasible by direct conversion of fibroblasts to renal tubular cells [e.g., using transcription factors, FOXA3, HNF1A, HNF4A (Kaminski et al., 2016)] or hepatocytes [e.g., using transcription factors, ATF5, PROX1, FOXA2, FOXA3, HNF4A (Huang et al., 2014)]. These experiments are even potentially scalable to a population-wide basis (Chung et al., 2015). However, replacing pharmacokinetic data acquirable from live animal experimentation would require complex 3D modeling. This is currently extremely challenging and will be an important development for the future.

### *In situ* Transdifferentiation

Innovative research has explored transdifferentiation approaches *in situ* that could be implemented to address cell loss or dysfunction in various diseases. Examples of this have included conversion of: pancreatic exocrine cells to pancreatic beta cells (Zhou et al., 2008), as potential therapeutic approaches for diabetes; cardiac fibroblasts to cardiomyocyte-like cells (Mathison et al., 2017) for myocardial infarction; astrocytes to neurons for neurodegenerative diseases, such as Parkinson’s disease, Alzheimer’s disease, Huntington’s disease, and amyotrophic lateral sclerosis (ALS) (Zhou et al., 2008; Guo et al., 2013; Mathison et al., 2017; Rivetti di Val Cervo et al., 2017; Qian et al., 2020; Wu et al., 2020).

Transdifferentiation offers a promising avenue for replacement of neural cells that are lost in neurological conditions. Current lines of evidence for *in situ* transdifferentiation for neurological conditions include: (i) the overexpression of NeuroD1, Ascl1 and Lmx1a, and the microRNA miR218 successfully converted striatal astrocytes into dopaminergic neurons in a Parkinson’s disease mouse model (Rivetti di Val Cervo et al., 2017). (ii) The repression of the RNA binding protein, polypyrimidine tract binding protein (PTB), converted midbrain astrocytes to dopaminergic neurons in the mouse brain and re-established the nigrostriatal circuit in a Parkinson’s disease model (Qian et al., 2020). (iii) The astrocyte-targeted overexpression of NeuroD1 in an Alzheimer’s disease mouse model at 7 and 14 months old converted reactive cortical astrocytes into glutamatergic and GABAergic neurons (Guo et al., 2013). (iv) The combined activation of NeuroD1 and Dlx2 in two different Huntington’s disease mouse models converted striatal astrocytes to GABAergic neurons with 80% efficiency and > 50% of converted cells classed as medium spiny neurons (Wu et al., 2020). (v) The overexpression of Sox10 alone in demyelinated mouse brains converted astrocytes into oligodendrocyte-like cells (Mokhtarzadeh Khanghahi et al., 2018). (v) CRISPR activation of Ngn2 and Isl1 using AAVs in mouse spinal cord converted 43% of astrocytes to motor neurons at 42 days post infection (Zhou et al., 2021), offering a promising avenue for neuronal replacement in ALS or spinal cord injury. Collectively, these studies highlight the promising advances of *in situ* transdifferentiation for neural cell replacement in neurological conditions. Further work is required to understand the potential long-term ramifications of transdifferentiation *in vivo*.

Understanding the impacts of the disease phenotypes on transdifferentiated cells and the immune responses to transdifferentiation *in vivo* need to be further explored. This is especially the case given the challenges of spatial, temporal and dose control in human patients that would differ vastly from animal studies. In particular, the effect of *in vivo* transdifferentiation of glial cells to neurons, potentially depleting the glial cell population and disrupting homeostasis. In addition, transdifferentiated cells contain the same disease-related characteristics as the host cells that are being replaced (e.g., genetic risk factors, protein aggregates, inflammatory signals), thus the long-term viability and function of transdifferentiated cells should be examined. Addressing aberrant genetic mutations may also require *in situ* genetic correction. *In situ* conversion of genetic mutations in late-onset neurodegenerative diseases may be unrealistic during a patient’s lifetime or disease course. Meanwhile, targeted delivery of transcription factors or small molecules across the blood brain barrier and into glial cells may be challenging and lentiviral or adenoviral delivery methods may result in dose dependent partial or unpredictable transdifferentiated cell types. Nonetheless, this preliminary research provides a promising avenue to understand and treat neurological diseases.

### Final Comments

For specific transdifferentiation outcomes of neurologically relevant cell types, rigorous optimization of dose-response and duration of a range of small molecules, growth factors, cytokines and transcription factors should be explored. The potential benefit of transdifferentiation protocols includes rapid and efficient generation of live cell culture models with characteristics of aging. With further development, transdifferentiation methods offer a valuable avenue toward animal-free, larger sample size, human live cell model studies for neurological cell pathology and pharmacogenetic research.

## Author Contributions

All authors listed have made a substantial, direct, and intellectual contribution to the work, and approved it for publication.

## Conflict of Interest

The authors declare that the research was conducted in the absence of any commercial or financial relationships that could be construed as a potential conflict of interest.

## Publisher’s Note

All claims expressed in this article are solely those of the authors and do not necessarily represent those of their affiliated organizations, or those of the publisher, the editors and the reviewers. Any product that may be evaluated in this article, or claim that may be made by its manufacturer, is not guaranteed or endorsed by the publisher.
